# Necessity of Sleep for Motor Gist Learning in Mice

**DOI:** 10.3389/fnins.2019.00293

**Published:** 2019-04-05

**Authors:** Ward D. Pettibone, Korey Kam, Rebecca K. Chen, Andrew W. Varga

**Affiliations:** Mount Sinai Integrative Sleep Center, Division of Pulmonary, Critical Care, and Sleep Medicine, Icahn School of Medicine at Mount Sinai, New York, NY, United States

**Keywords:** sleep disruption, generalization, rotarod, flexibility, natural wake

## Abstract

With respect to behavior, the term memory “consolidation” has canonically been used to describe increased fidelity during testing to a learned behavior shaped during training. While the sleeping brain appears to certainly aid in consolidation by this definition for a variety of memories, including motor memories, growing evidence suggests that sleep allows for much more flexible use of the information encountered during prior wakefulness. Sleep has been shown to augment the extraction of gist or patterns from wake experience in human subjects, but this has been difficult to recapitulate in animal models owing to the semantic requirements in many such tasks. Here we establish a model of motor gist learning in mice in which two bouts of exclusive forward running on the rotarod significantly augments the first experience of exclusive backward running. This augmentation does not occur if sleep is disrupted following the forward running template behavior or if a period of natural wakefulness follows one of the two bouts of exclusive forward running. This suggests that sleep is required for the extraction of the motor gist of forward running to apply to backward running.

## Introduction

A benefit of sleep on motor learning has been demonstrated in a variety of tasks in human subjects. For example, when trained on a finger-tapping motor sequence task, subjects improve overnight with no further practice (Fischer et al., 2002; Walker et al., 2002). Additionally, increased complexity of the task leads to greater gains in learning – in particular, the gains after learning a five-element unimanual sequence are lower compared with the gains after learning a nine-element bimanual sequence (Kuriyama et al., 2004). When sleep is disrupted with obstructive sleep apnea, offline gains in motor performance are not as robust (Djonlagic et al., 2012). Acute sleep disruption following motor task acquisition has been demonstrated to impair the subsequent offline gains following rodent motor learning tasks such as the rotarod (Yang et al., 2014) and skilled reaching task (Varga et al., 2014a). As in humans, increasing motor task complexity, for example by learning to run on a complex wheel, appears to augment the offline benefit of sleep (Nagai et al., 2016).

In these examples, the term sleep-dependent memory “consolidation” might seem appropriate. Presumably the sleeping brain has the capacity to analyze and parse prior wake motor trials and cement the ones that were completed with optimal performance. However, sleep is involved in more than just consolidation. Newly learned memories can be integrated into pre-existing paradigms allowing for enhanced assimilation and utility. Human studies have shown that sleep augments the extraction of the general idea of a task, or its “gist” whereby the brain identifies common features of new wake experiences and incorporates them into a new schema (Payne et al., 2009; Lutz et al., 2017).

Such gist learning, or generalization, can be applied to motor learning. The most commonly studied form of this is inter-manual transfer, where motor learning in one limb can augment subsequent motor performance on the same task in the contralateral untrained limb (Censor, 2013; Veldman et al., 2017). Such ability is thought to be augmented by a period of sleep after the initial motor learning (Witt et al., 2010), but this has not been investigated extensively, and nearly all studies of this phenomenon have occurred in human subjects. Establishing a mouse model of motor gist learning would be important for evaluating potential molecular and electrophysiological mechanisms used by the brain to elicit such plasticity, including potential sleep-state specific effects. In the current study, we employ one of the most commonly used tests of motor learning in rodents, the rotarod test of motor coordination, in a paradigm in which mice were trained on a rotarod running exclusively forward, and then tested running exclusively backward, with either *ad libitum* or disrupted sleep between training and testing. The results indicate that motor gist learning is possible in a mouse model and that sleep is critical for this learning to take place.

## Materials and Methods

### Animals

Adult wild type C57BL/6 male mice (3–6 months of age) were kept on a 12 h/12 h light/dark schedule with lights on at 9:00 AM [zeitgeber time (ZT) 0]. Mice were group housed (3–5 mice per cage). Food and water were available *ad libitum*. All experiments were approved by the Institution of Animal Care and Use Committee of the Icahn School of Medicine at Mount Sinai and were carried out in accordance with all National Institutes of Health guidelines.

### Surgery

Surgical and implantation procedures were performed as previously described (Kam et al., 2016). Mice were anesthetized continuously with inhaled isoflurane and placed in a stereotaxic apparatus (David Kopf). After exposing the skull, five electrodes were positioned. Two subdural electrodes (2.5 mm diameter screws with tapered tips, Pinnacle Technologies), symmetrically placed over left and right primary motor cortices (1.5 mm anterior to Bregma, ± 2.0 mm lateral to the midline) served as EEG electrodes. Two epidural screw electrodes were placed above the cerebellum to serve as reference and ground. A bipolar, twisted stainless steel electrode (California Fine Wire Co.) inserted into the nuchal muscles served as an electromyogram (EMG) site. After implantation, a 6-pin connector (Mill-max) was centered over the skull with dental cement (Dentsply) and the animal was placed in its home cage on top of a heating pad set to 37°C (Harvard Apparatus) until fully ambulatory. All animals were supplied with subcutaneous hydration and pain control (buprenorphine) following surgery.

### Sleep Recordings and Sleep Disruption

#### EEG/EMG Data Acquisition

Mice were housed individually for at least a week after surgery in the room where EEG was conducted so that they would acclimate to the recording environment. A 24-h tethered session served as acclimation prior to sleep recordings. Recordings were performed in a cylindrical chamber with a ∼12 inch base coupled with a multichannel commutator (Pinnacle Technologies) to allow freedom of movement with access to food pellets and water over the 24-h recording session.

Signals were acquired at 1000 Hz sampling rate and bandpass filtered from 0.5–100 Hz (Pinnacle). Simultaneous video was recorded continuously at 10 frames per second (synchronized with the EEG record) during both light and dark periods using an infrared LED camera with Sirenia Video Acquisition software (Pinnacle).

#### EEG/EMG Data Analysis

Data analysis was performed using MATLAB (MathWorks) with the Statistics/Signal Processing toolboxes and the FieldTrip toolbox (Oostenveld et al., 2011).

#### Sleep-Wake Analysis

Sleep/wake scoring was performed as previously described (Kam et al., 2016). Video-EEG/EMG was analyzed continuously to characterize behavioral states in 1 s epochs. Behavioral states (wakefulness, NREM and REM sleep) were classified in an automated epoch-free approach based on these criteria with subsequent visual inspection and manual editing as needed:

• Time-varying ratio of theta over delta power (θ, 5–10 Hz; δ, 1–4 Hz) using both the right and left primary motor cortex lead.
• Presence of slow waves (delta power, 1–4 Hz) defined as segments with greater than 1 *z*-score analyzed from both the right and left primary motor cortex lead across the entire recording.
• Movement, detected by EMG and confirmed by simultaneous manual review of video.

REM sleep was defined by a high ratio of theta/delta power (ratio >2.5), and little or no movement of the body (based on EMG < 1 *z*-score). In addition, a criterion for REM sleep was that the prior behavioral state was NREM sleep (which is the normal pattern for sleep in rodents). REM sleep segments separated by less than 3 s were merged because these were periods when small twitches or slight posture changes appeared to interrupt an otherwise continuous period of REM sleep. If movement was <1 *z*-score, but other criteria for REM were not met, the behavioral state was classified as NREM sleep or quiet wakefulness. NREM was discriminated from quiet wakefulness based on power in the delta band and presence of putative spindles. Thus, NREM sleep showed a lower ratio of theta/delta power (<2.5) than quiet wakefulness. Sleep episodes were confirmed manually by reviewing video and finding that mice lay in a curled body position. Periods with relatively low delta power (<1 *z*-score) and minimal movement (<1 *z*-score) were designated as quiet wakefulness. Periods with movement for >3 s were classified as active wakefulness and included exploration/walking, grooming, sniffing, consummatory behavior (eating/drinking), and arousals from sleep (both spontaneous and induced by the sleep disruption chamber). All spectral thresholds were verified manually for each recording.

Continuity of behavioral states was assessed using a resampling cumulative distribution approach (Kishi et al., 2011; Varga et al., 2014b).

#### Sleep Disruption (SD)

Animals were placed in a custom designed chamber with a ∼12 inch diameter slowly rotating round floor in which wires forming an “X” hung about 1 cm above the floor. During sleep, a somatosensory stimulus created by the motionless mouse meeting the wire occurred automatically once every 10 s. Food and water were available *ad libitum*. Sleep disruption occurred during the light phase between ZT2 and ZT12.

### Rotarod Task

Mice completed 10 consecutive trials on a rotarod (Ugo Basile), accelerating from 4 to 40 RPM over 5 min, with a 3 min inter-trial interval. Each trial terminated when the mouse fell off the rod, when the animal clung to the rod for a full 360 degree rotation, or when the maximum time of 300 s was reached, and this latency was recorded. To prevent mice from turning around and thereby ensuring exclusive forward or exclusive backward running, corrugated plastic dividers were inserted over the rod to narrow the available space. All rotarod training took place between ZT 0 and ZT 2.

#### Statistics

Data were analyzed using SigmaPlot version 11.0 and Matlab (R 2018b). Comparisons between sleep physiology variables were performed using paired *t*-tests for normally distributed data and mean values +/- the standard error of the mean are reported. Sleep continuity was assessed as duration of sleep runs, defined as the duration of consecutive epochs of sleep scored as non-REM or REM sleep, terminated by one or more epochs scored as another stage, including wake. Kolmogorov-Smirnov tests were used to compare disrupted versus *ad libitum* sleep conditions on the survival curves.

For the forward running template behavior, two primary variables were assessed, which included performance during the first 3 trials of session 2 normalized to the last 3 trials of session 1 (F3S2/L3S1) and the mean performance of all trials in session 2 normalized to the mean performance in all trials of session 1 (Mean S2/S1). Comparison between groups was completed with a one-way ANOVA on ranks as these data were not normally distributed.

For the backward running gist behavior, primary analysis included a mixed factorial repeated measures ANOVA, which allowed assessment of a main effect of trial number (e.g., general within-session learning), a main effect of sleep condition, and interactions between these two. Secondary analysis included one-way ANOVA on ranks of mean performance across all backward running trials per mouse between sleep conditions with Dunn’s *post hoc* comparisons to the naïve condition as the control.

## Results

### Motor Gist Learning Is Possible in a Mouse

**FIGURE 1:**
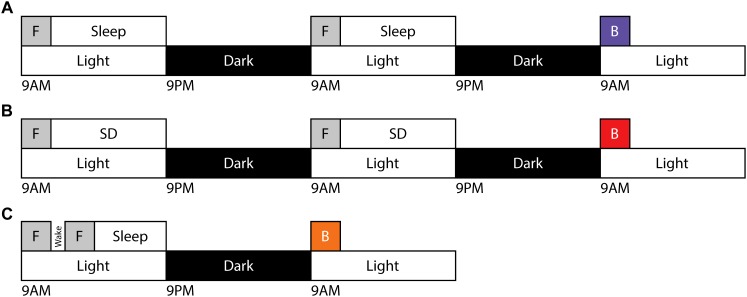
Training and testing paradigms for motor gist learning. Timeline demonstrating the exclusive forward running template behavior [***F***] occurring beginning at ZT0 on two consecutive days followed by *ad libitum* sleep **(A)** or 10 h of acute sleep disruption **(B)**. Testing on exclusive backward running [***B***] occurs beginning at ZT0 on the third day. **(C)** Timeline demonstrating the exclusive forward running template behavior [***F***] occurring twice on day 1 beginning at ZT0 separated by 30 min of natural wake prior to testing on exclusive backward running [***B***] beginning at ZT0 on day 2.

**FIGURE 2:**
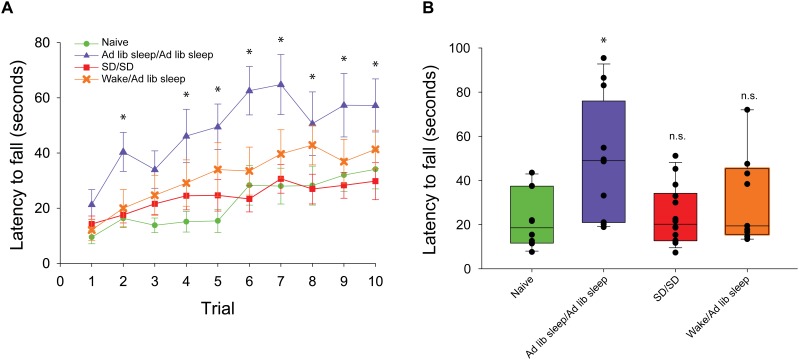
Rotarod performance during exclusive backward running. **(A)** Latency to fall is measured across 10 consecutive trials. Mice experiencing exclusive forward running template learning over 2 days followed by *ad libitum* sleep (blue triangle, *n* = 12) demonstrated significantly better backward running performance than mice experiencing exclusive forward running template learning over 2 days followed by 10 h of sleep disruption (SD) (red square, *n* = 14), mice experiencing exclusive forward running template learning separated by 30 min of natural wake prior to *ad libitum* sleep (orange X, *n* = 9), or mice naïve to running exclusively backward (green circle, *n* = 10). **(B)** Box plots showing the average exclusive backward running performance over 10 trials between sleep conditions. ^∗^*p* < 0.05, n.s. = not significant.

In order to evaluate the capacity for motor gist learning, mice were trained to run exclusively forward for 10 trials per day for 2 consecutive days between ZT 0 and ZT 2 with subsequent *ad libitum* sleep before running exclusively backward for the first time on the 3rd consecutive day (Figure 1A). Backward performance in this case was significantly augmented compared to mice that were handled and placed on the rotarod but did not run on the first 2 days followed by running exclusively backward for the first time on the 3rd consecutive day (naïve) (Figure 2).

### Effects of Automated Sleep Disruption on Sleep Architecture

**FIGURE 3:**
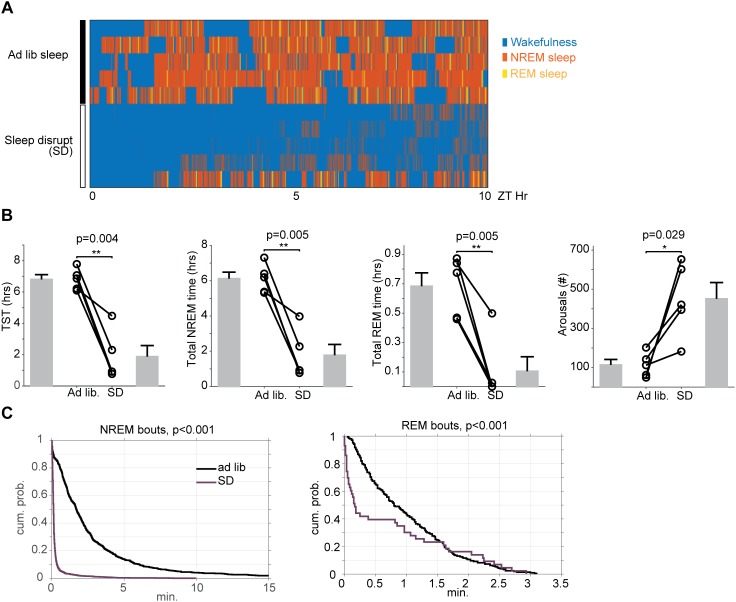
Effects of 10 h of automated sleep disruption on sleep physiology. **(A)** Hypnograms between ZT0 and ZT10 for five mice during baseline undisrupted sleep (top) and during 10 h of sleep disruption (bottom). **(B)** Mice undergoing 10 h of sleep disruption show significant decreases in total sleep time, NREM duration, and REM duration, and significant increases in wake duration and number of state transitions. **(C)** Survival curves (cumulative probability distributions) of NREM and REM runs in the normal baseline sleep and sleep disrupted conditions demonstrate significant fragmentation of all vigilance states. The curves represent the probability (*y*-axis) of sleep of that stage lasting the duration shown on the *x*-axis. Leftward shifted curves reflect higher sleep fragmentation. ^∗^*p* < 0.05, ^∗∗^*p* < 0.01.

In order to ascertain the impact of automated sleep disruption on sleep physiology, a subset of mice (*n* = 5) were surgically implanted with EEG/EMG and video recorded during a period of either *ad libitum* sleep or ongoing sleep disruption between ZT 2 and ZT 12. Total sleep time was reduced on average 73% (112 ± 42 min for SD vs. 408 ± 18 min for *ad libitum* sleep, paired *t*-test, *p* = 0.004) with decreases in both non-REM sleep (106 ± 37 min for SD vs. 367 ± 22 min for *ad libitum* sleep, paired *t*-test, *p* = 0.005) and REM sleep (6 ± 6 min for SD vs. 41 ± 5 min for *ad libitum* sleep, paired *t*-test, *p* = 0.005). The number of arousals during sleep was also significantly increased with sleep disruption (451 ± 84 arousals for SD vs. 113 ± 28 min for *ad libitum* sleep, paired *t*-test, *p* = 0.029) (Figure 3A,B). Sleep was not only reduced, but also significantly fragmented, as evidenced by significant leftward shifts in the cumulative duration probability distribution of both non-REM (Kolmogorov-Smirnov test, *p* < 0.001) and REM sleep (Kolmogorov-Smirnov test, *p* < 0.001) during SD (Figure 3C), indicating that sleep occurred in smaller bouts than during baseline *ad libitum* sleep.

### Acute Sleep Disruption Impairs Motor Gist Learning

In order to evaluate a role for sleep in the extraction of the gist of motor task, mice ran exclusively forward on the rotarod for two consecutive days between ZT 0 and ZT 2, and then experienced acute sleep disruption for 10 h (ZT 2–12) afterward on both days (Figure 1B). On the third day, they ran exclusively backward for the first time. Latency to fall was lower as compared with the mice that had been allowed to sleep *ad libitum*, and comparable to that of the naïve cohort.

### Natural Wake After Acquisition of First 10 Trials of Template Learning Is Insufficient to Impart the Benefit of Motor Gist Extraction

An alternative method to address the requirement of sleep for possible motor gist learning is to evaluate the effect of a period of normal wakefulness following learning of the template behavior. Mice will not remain naturally awake for extended periods of time, but will reliably remain awake for at least 30 min following rotarod learning. Therefore, we investigated a paradigm in which mice ran exclusively forward on the rotarod for 10 trials, followed by a 30-min break during which mice were visually confirmed to maintain wake. Mice were trained for an additional 10 trials running exclusively forward before being allowed to sleep *ad libitum* until ZT 0 the following morning, at which point they were tested running exclusively backward for the first time (Figure 1C). Thus, mice experienced the same total number of forward template trials (20) as in the prior paradigms. The benefit of this paradigm on initial exclusively backward running was reduced in comparison to the paradigm containing sleep after both sets of exclusively forward running template trials. Performance of individual mice on exclusively backward running across trials for each sleep condition is shown in Supplementary Figure S1.

Two-way repeated measures ANOVA analysis of all conditions demonstrated that there was a main effect of trial number (*F*_(1,9)_ = 24.0, *p* < 0.001), main effect of sleep condition (*F*_(1,3)_ = 6.2, *p* = 0.003) and significant interaction between trial number and condition (*F*_(2,27)_ = 1.9, *p* = 0.009). *Post hoc* analysis of individual trials demonstrated that only performance in the *ad libitum* sleep condition following forward running was significantly improved vs. the naïve condition during trials 2 and 4–10. No individual trials were significantly different from the naïve condition in the sleep disrupted and natural wake conditions (Figure 2A). Assessment of mean performance per mouse during backward running showed a significant difference between sleep condition groups (*H* = 9.85, *p* = 0.02, ANOVA on ranks) with *post hoc* comparisons against the naïve condition as a control showing only the *ad libitum* sleep condition was significantly improved (Figure 2B).

### Sleep Condition Between Template Behavior Forward Running Sessions Does Not Impact Offline Change in Forward Running Performance

**FIGURE 4:**
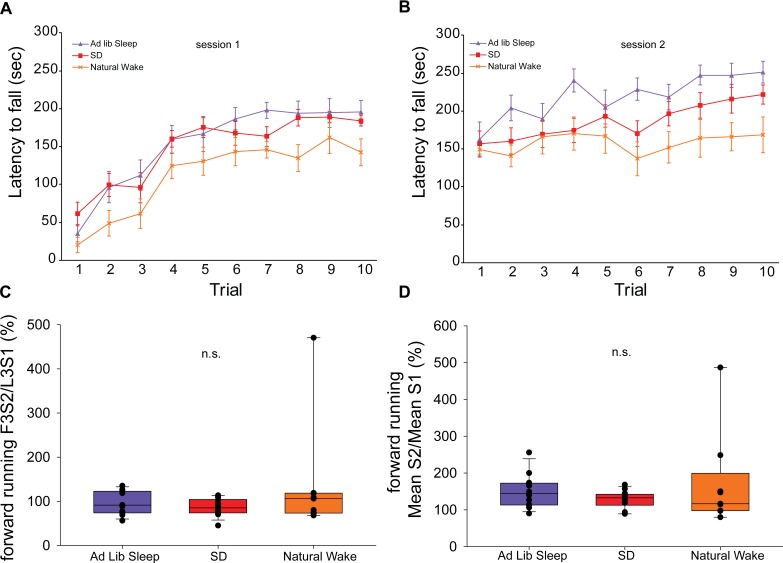
Rotarod performance during exclusive forward running template behavior. Latency to fall on 10 consecutive trials of the exclusive forward running template behavior during session 1 **(A)** and session 2 **(B)**. Measures of offline change in performance in the first 3 trials of session 2 normalized to performance in the last 3 trials of session 1 (F3S2/L3S1) **(C)** as well as the mean performance in session 2 normalized to the mean performance in session 1 (mean S2/mean S1) **(D)** show no effect of sleep condition.

Forward running performance during the template behavior in sessions 1 and 2 for animals in each sleep condition is shown in Figure 4, panels A and B, respectively. In order to assess how the experience of *ad libitum* sleep, acute SD, or natural wake impacted offline change in the forward running template behavior we evaluated performance in the first 3 trials of session 2 normalized to performance in the last 3 trials of session 1 (F3S2/L3S1) (Figure 4C) as well as the mean performance in session 2 normalized to the mean performance in session 1 (mean S2/mean S1) (Figure 4D). By both of these metrics, no significant differences in offline change were observed (F3S2/L3S1: *H* = 1.19, *p* = 0.55; mean S2/mean S1: *H* = 1.29, *p* = 0.53, ANOVA on ranks).

## Discussion

The emerging thinking on the role of sleep in memory is that it is involved in the processing or evolution of memories created during prior wake experience (Stickgold and Walker, 2013). Certainly this can include the “consolidation” of memories, but sleep also appears to promote flexible incorporation of the wake experience into existing schemas enabling rule learning, pattern recognition, and forms of generalization including gist extraction. Gist learning has been demonstrated in human subjects for tasks such as word list learning (Payne et al., 2009) and visual perceptual tasks (Lutz et al., 2017), but the verbal feedback required for these tests makes them difficult to implement in animals. To circumvent this limitation in a mouse model, we implemented a commonly used motor learning task, the rotarod, to evaluate performance on a related motor coordination behavior (exclusive backward running) after learning a template behavior (exclusive forward running), and with this novel paradigm we demonstrate for the first time that mice are capable of gist learning. To our knowledge, this is the first example of gist learning in an animal model. We should note that the ability to generalize or abstract gist from wake experience may take variable amounts of time, and in some cases, long periods of time, but that this ability likely depends on both the complexity of the initial learning/encoding experience and the degree of abstraction required. We suspect that the degree of abstraction required from exclusive forward running as applied to exclusive backward running is not likely to be high, and as such the gist effect can be observed somewhat acutely.

Sleep appears to be required for the extraction of the motor gist following forward running, as when sleep is acutely disrupted following exclusive forward running, the augmentation in exclusive backward running is not observed. A reasonable criticism of this interpretation is that it may be stress, rather than absence of sleep *per se*, that is a contributing factor, and chronic systemic corticosterone injections have been shown to impair standard rotarod learning in mice (Harle et al., 2017). While we cannot completely rule out this possibility, it is worth noting that plasma corticosterone levels were not elevated following a related sleep disruption paradigm lasting 20 h in rats (Roman et al., 2005). Additionally, inhibition of corticosterone signaling with either adrenalectomy (Ruskin et al., 2006) or systemic metyrapone administration (Tiba et al., 2008) did not impact the deleterious effect of sleep disruption on spatial learning or fear conditioning learning, respectively. Furthermore, stress can even augment, rather than inhibit, certain forms of motor learning (Hordacre et al., 2016; Littmann and Shields, 2016).

To avoid the potential limitations of the stress response on the behavioral manifestation of learning, paradigms can be employed in which the effect of natural wake following initial learning is evaluated. This type of paradigm is common in human subjects where initial training can be done in the morning with re-testing occurring in the evening after a period of normal wakefulness, although it suffers from alternative confounds, such as any circadian effects on initial encoding. Using such a paradigm, it has been demonstrated that offline gains in a finger tapping motor sequence task are greater across a period of sleep versus a period of normal daytime wakefulness (Fischer et al., 2002). Employing an equivalent paradigm in rodents, however, is not straightforward as rodents do not maintain a single vigilance state (sleep or wake) for extended periods of time across both nocturnal and diurnal periods. In the current work, we demonstrate that when the initial template behavior (exclusive forward running) is followed by a 30 min period of natural wake, motor gist learning does not occur, even if *ad libitum* sleep occurs following the second exposure to the template behavior.

It is possible that this observation is the result of insufficiently spaced training, irrespective of the vigilance state following the training. It has long been observed that spaced training can improve learning efficiency compared to massed training for many types of learning behaviors (Cepeda et al., 2008; Litman and Davachi, 2008), however, the extent to which this is true to motor learning has primarily been investigated in human subjects. Results have been mixed, with some studies showing a benefit of spaced training on motor learning (Kwon et al., 2015; Spruit et al., 2017) and some showing no benefit versus massed training (Nadeau et al., 2014; Wiseheart et al., 2017). The effect of spaced versus massed training and has never clearly been formally assessed for standard rodent rotarod learning, let alone motor gist learning. It bears noting that the effects of spaced training are dependent on several factors in addition to the specific learning behavior, including duration between training trials, the duration between the final training trial and the testing trial, and whether sleep occurs during any of these offline periods. Thus, while it may be that two template exclusive forward running behaviors separated by 30 min is less efficient for motor gist extraction than template behaviors separated by 24 h irrespective of vigilance state, we feel the current most parsimonious interpretation of the behavior is that natural wakefulness following one template behavior is insufficient to impart the ability to extract the motor gist from that template behavior. Future experiments will be required to better understand the individual contributions of training spacing and sleep in motor gist learning.

It is noteworthy that the various sleep conditions did not significantly impact offline behavioral change during the exclusive forward running template behavior by two different measures: the first 3 trials of session 2 normalized to the last 3 trials of session 1 (F3S2/L3S1) and the mean performance of all trials in session 2 normalized to the mean performance of all trials in session 1 (mean S2/mean S1). This finding differs from some prior studies examining offline change in rotarod performance in mice (Yang et al., 2014; Pettibone et al., 2017), but is consistent with another study where a benefit of sleep was only observed when the task was made more challenging by using a complex wheel but not on a standard rotarod (Nagai et al., 2016). Such differences may be related to any of a variety of variables in the rotarod task itself, such as individual trial duration, number of trials per session, inter-trial interval, and degree of rod acceleration, as well as variables such as the age and sex distribution of the mice. In any case, the current observations suggest that motor gist learning is more sensitive to sleep disruption than canonical motor learning consolidation.

Overall, our results expand the current understanding of the types of learning and memory behaviors impacted by sleep disruption, which bears relevance to a variety of clinical sleep disorders characterized by sleep fragmentation. Furthermore, the current behavioral paradigm of motor gist learning in mice can serve as a template for the delineation of neural circuits enabling higher order cognitive processes.

## Author Contributions

AV designed the study. WP, KK, and RC performed the experiments and acquired the data. AV, WP, and KK analyzed the data and wrote the manuscript.

## Conflict of Interest Statement

The authors declare that the research was conducted in the absence of any commercial or financial relationships that could be construed as a potential conflict of interest.

## Supplementary Material

FIGURE S1 Individual traces of rotarod performance during exclusive backward running. Gray lines show the individual performance traces and colored lines show the averaged performance for the naïve, *ad libitum* sleep, sleep disruption (SD), and natural wake conditions.

## References

[B1] Censor N. (2013). Generalization of perceptual and motor learning: a causal link with memory encoding and consolidation? *Neuroscience* 250 201–207. 10.1016/j.neuroscience.2013.06.062 23850685 PMC3787068

[B2] Cepeda N. J. Vul E. Rohrer D. Wixted J. T. Pashler H. (2008). Spacing effects in learning: a temporal ridgeline of optimal retention. *Psychol. Sci.* 19 1095–1102. 10.1111/j.1467-9280.2008.02209.x 19076480

[B3] Djonlagic I. Saboisky J. Carusona A. Stickgold R. Malhotra A. (2012). Increased sleep fragmentation leads to impaired off-line consolidation of motor memories in humans. *PLoS One* 7:e34106. 10.1371/journal.pone.0034106 22470524 PMC3314699

[B4] Fischer S. Hallschmid M. Elsner A. L. Born J. (2002). Sleep forms memory for finger skills. *Proc. Natl. Acad. Sci. U.S.A.* 99 11987–11991. 10.1073/pnas.182178199 12193650 PMC129381

[B5] Harle G. Lalonde R. Fonte C. Ropars A. Frippiat J. P. Strazielle C. (2017). Repeated corticosterone injections in adult mice alter stress hormonal receptor expression in the cerebellum and motor coordination without affecting spatial learning. *Behav. Brain Res.* 326 121–131. 10.1016/j.bbr.2017.02.035 28263830

[B6] Hordacre B. Immink M. A. Ridding M. C. Hillier S. (2016). Perceptual-motor learning benefits from increased stress and anxiety. *Hum. Mov. Sci.* 49 36–46. 10.1016/j.humov.2016.06.002 27309494

[B7] Kam K. Duffy A. M. Moretto J. LaFrancois J. J. Scharfman H. E. (2016). Interictal spikes during sleep are an early defect in the Tg2576 mouse model of beta-amyloid neuropathology. *Sci. Rep.* 6:20119. 10.1038/srep20119 26818394 PMC4730189

[B8] Kishi A. Natelson B. H. Togo F. Struzik Z. R. Rapoport D. M. Yamamoto Y. (2011). Sleep-stage dynamics in patients with chronic fatigue syndrome with or without fibromyalgia. *Sleep* 34 1551–1560. 10.5665/sleep.1396 22043126 PMC3198210

[B9] Kuriyama K. Stickgold R. Walker M. P. (2004). Sleep-dependent learning and motor-skill complexity. *Learn. Mem.* 11 705–713. 10.1101/lm.76304 15576888 PMC534699

[B10] Kwon Y. H. Kwon J. W. Lee M. H. (2015). Effectiveness of motor sequential learning according to practice schedules in healthy adults; distributed practice versus massed practice. *J. Phys. Ther. Sci.* 27 769–772. 10.1589/jpts.27.769 25931727 PMC4395711

[B11] Litman L. Davachi L. (2008). Distributed learning enhances relational memory consolidation. *Learn. Mem.* 15 711–716. 10.1101/lm.1132008 18772260

[B12] Littmann A. E. Shields R. K. (2016). Whole body heat stress increases motor cortical excitability and skill acquisition in humans. *Clin. Neurophysiol.* 127 1521–1529. 10.1016/j.clinph.2015.11.001 26616546 PMC4747790

[B13] Lutz N. D. Diekelmann S. Hinse-Stern P. Born J. Rauss K. (2017). Sleep supports the slow abstraction of gist from visual perceptual memories. *Sci. Rep.* 7:42950. 10.1038/srep42950 28211489 PMC5314355

[B14] Nadeau S. E. Davis S. E. Wu S. S. Dai Y. Richards L. G. (2014). A pilot randomized controlled trial of D-cycloserine and distributed practice as adjuvants to constraint-induced movement therapy after stroke. *Neurorehabil. Neural Repair* 28 885–895. 10.1177/1545968314532032 24769437

[B15] Nagai H. de Vivo L. Bellesi M. Ghilardi M. F. Tononi G. Cirelli C. (2016). Sleep consolidates motor learning of complex movement sequences in mice. *Sleep* 40:zsw059. 10.1093/sleep/zsw059 28364506 PMC6084756

[B16] Oostenveld R. Fries P. Maris E. Schoffelen J. M. (2011). FieldTrip: open source software for advanced analysis of MEG, EEG, and invasive electrophysiological data. *Comput. Intell. Neurosci.* 2011:156869. 10.1155/2011/156869 21253357 PMC3021840

[B17] Payne J. D. Schacter D. L. Propper R. E. Huang L. W. Wamsley E. J. Tucker M. A. (2009). The role of sleep in false memory formation. *Neurobiol. Learn. Mem.* 92 327–334. 10.1016/j.nlm.2009.03.007 19348959 PMC2789473

[B18] Pettibone W. Kam K. Chen R. Ly A. Varga A. W. (2017). Interactions between sleep deprivation, motor learning and S6 kinase signaling. *Sleep* 40 A92–A92. 10.1093/sleepj/zsx050.249 PMC7315768 31608388

[B19] Roman V. Walstra I. Luiten P. G. Meerlo P. (2005). Too little sleep gradually desensitizes the serotonin 1A receptor system. *Sleep* 28 1505–1510. 16408408

[B20] Ruskin D. N. Dunn K. E. Billiot I. Bazan N. G. LaHoste G. J. (2006). Eliminating the adrenal stress response does not affect sleep deprivation-induced acquisition deficits in the water maze. *Life Sci.* 78 2833–2838. 10.1016/j.lfs.2005.11.003 16325867

[B21] Spruit E. N. Band G. P. H. van der Heijden K. B. Hamming J. F. (2017). The effects of spacing, naps, and fatigue on the acquisition and retention of laparoscopic skills. *J. Surg. Educ.* 74 530–538. 10.1016/j.jsurg.2016.11.003 27988169

[B22] Stickgold R. Walker M. P. (2013). Sleep-dependent memory triage: evolving generalization through selective processing. *Nat. Neurosci.* 16 139–145. 10.1038/nn.3303 23354387 PMC5826623

[B23] Tiba P. A. Oliveira M. G. Rossi V. C. Tufik S. Suchecki D. (2008). Glucocorticoids are not responsible for paradoxical sleep deprivation-induced memory impairments. *Sleep* 31 505–515. 10.1093/sleep/31.4.505 18457238 PMC2279753

[B24] Varga A. W. Kang M. Ramesh P. V. Klann E. (2014a). Effects of acute sleep deprivation on motor and reversal learning in mice. *Neurobiol. Learn. Mem.* 114 217–222. 10.1016/j.nlm.2014.07.001 25046627 PMC4143485

[B25] Varga A. W. Kishi A. Mantua J. Lim J. Koushyk V. Leibert D. P. (2014b). Apnea-induced rapid eye movement sleep disruption impairs human spatial navigational memory. *J. Neurosci.* 34 14571–14577. 10.1523/JNEUROSCI.3220-14.2014 25355211 PMC4212062

[B26] Veldman M. P. Maurits N. M. Nijland M. A. M. Wolters N. E. Mizelle J. C. Hortobágyi T. (2017). Spectral and temporal electroencephalography measures reveal distinct neural networks for the acquisition, consolidation, and interlimb transfer of motor skills in healthy young adults. *Clin. Neurophysiol.* 129 419–430. 10.1016/j.clinph.2017.12.003 29304417

[B27] Walker M. P. Brakefield T. Morgan A. Hobson J. A. Stickgold R. (2002). Practice with sleep makes perfect: sleep-dependent motor skill learning. *Neuron* 35 205–211. 10.1016/S0896-6273(02)00746-8 12123620

[B28] Wiseheart M. D’Souza A. A. Chae J. (2017). Lack of spacing effects during piano learning. *PLoS One* 12:e0182986. 10.1371/journal.pone.0182986 28800631 PMC5553926

[B29] Witt K. Margraf N. Bieber C. Born J. Deuschl G. (2010). Sleep consolidates the effector-independent representation of a motor skill. *Neuroscience* 171 227–234. 10.1016/j.neuroscience.2010.07.062 20691764

[B30] Yang G. Lai C. S. Cichon J. Ma L. Li W. Gan W. B. (2014). Sleep promotes branch-specific formation of dendritic spines after learning. *Science* 344 1173–1178. 10.1126/science.1249098 24904169 PMC4447313

