# Supplementary figures and images for: Necessity of Sleep for Motor Gist Learning in Mice
Source: Front Neurosci. 2019 Apr 5;13:293. doi: 10.3389/fnins.2019.00293 (PMC6459967; doi:10.3389/fnins.2019.00293)

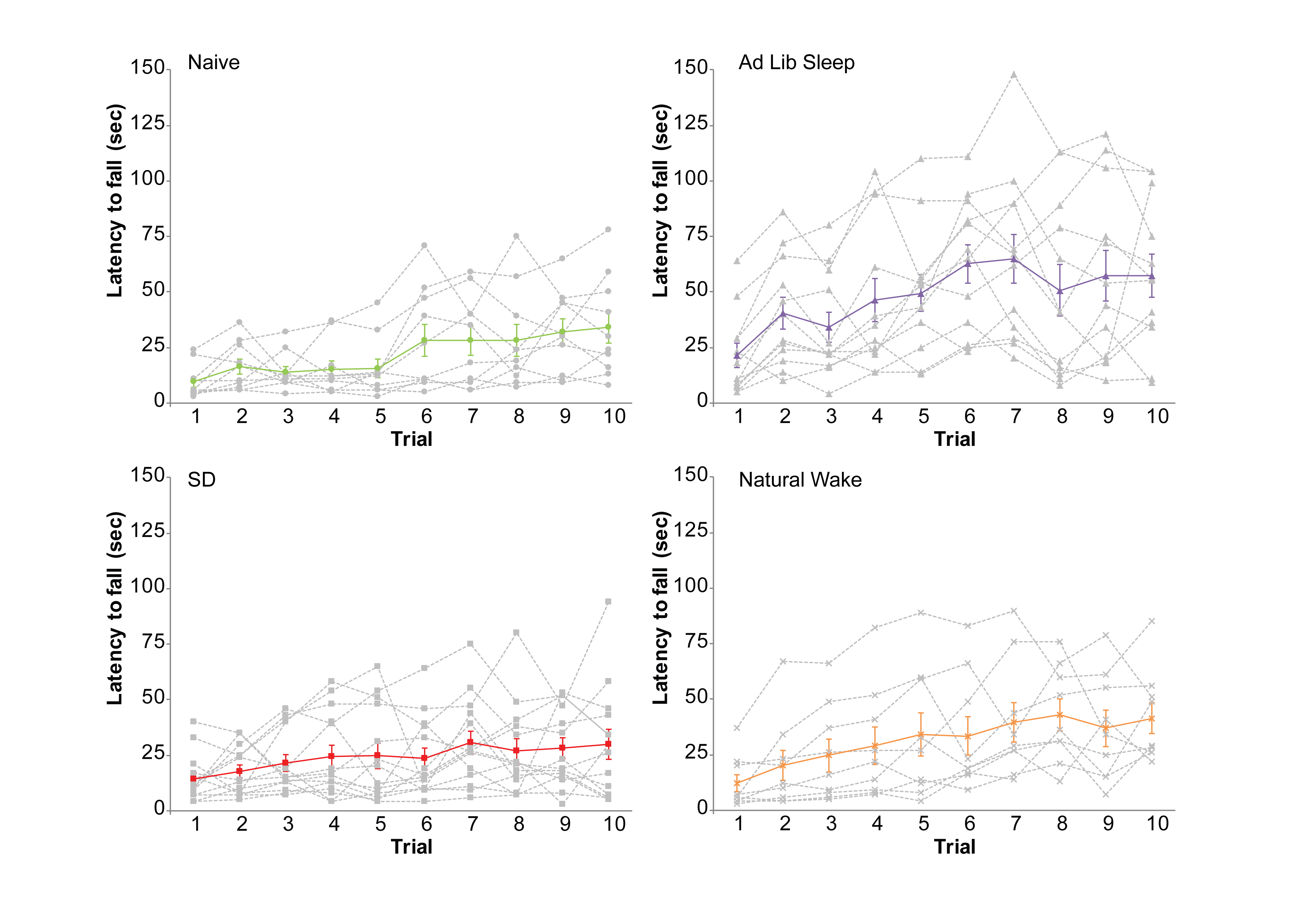

Supplement: FIGURE S1 — Individual traces of rotarod performance during exclusive backward running. Gray lines show the individual performance traces and colored lines show the averaged performance for the naïve, ad libitum sleep, sleep disruption (SD), and natural wake conditions. [file Image_1.TIF]
